# Genetic Characterization of Enterovirus A71 Circulating in Africa

**DOI:** 10.3201/eid2404.171783

**Published:** 2018-04

**Authors:** Maria Dolores Fernandez-Garcia, Romain Volle, Marie-Line Joffret, Serge Alain Sadeuh-Mba, Ionela Gouandjika-Vasilache, Ousmane Kebe, Michael R. Wiley, Manasi Majumdar, Etienne Simon-Loriere, Anavaj Sakuntabhai, Gustavo Palacios, Javier Martin, Francis Delpeyroux, Kader Ndiaye, Maël Bessaud

**Affiliations:** Institut Pasteur, Dakar, Senegal (M.D. Fernandez-Garcia, O. Kebe, K. Ndiaye);; Institut Pasteur, Paris, France (R. Volle, M.-L. Joffret, E. Simon-Loriere, A. Sakuntabhai, F. Delpeyroux, M. Bessaud);; Institut National de la Santé et de la Recherche Médicale, U994, Paris (R. Volle, M.-L. Joffret, F. Delpeyroux);; Centre Pasteur, Yaoundé, Cameroon (S.A. Sadeuh-Mba);; Institut Pasteur, Bangui, Central African Republic (I. Gouandjika-Vasilache);; University of Nebraska Medical Center, Omaha, Nebraska, USA (M.R. Wiley);; US Army Medical Research Institute of Infectious Diseases, Frederick, Maryland, USA (M.R. Wiley, G. Palacios);; National Institute for Biological Standards and Control, Hertfordshire, UK (M. Majumdar, J. Martin)

**Keywords:** enterovirus, acute flaccid paralysis, Africa, recombination, phylogenetic analysis, enterovirus A71, whole-genome analysis, viruses

## Abstract

We analyzed whole-genome sequences of 8 enterovirus A71 isolates (EV-A71). We confirm the circulation of genogroup C and the new genogroup E in West Africa. Our analysis demonstrates wide geographic circulation and describes genetic exchanges between EV-A71 and autochthonous EV-A that might contribute to the emergence of pathogenic lineages.

Enterovirus A71 (EV-A71; species *Enterovirus A*, genus *Enterovirus*, family *Picornaviridae*) is a common etiologic agent of hand, foot and mouth disease in young children. In addition, EV-A71 has been associated with severe and sometimes fatal neurologic diseases, including aseptic meningitis, encephalitis, and poliomyelitis-like acute flaccid paralysis (AFP) (*1*,*2*).

EV-A71 is classified info 7 genogroups (A–G). Genogroup A includes the prototype strain BrCr that was isolated in the United States in 1969 (*1*,*2*). Most EV-A71 isolates belong to genogroups B or C, which are each further divided into subgenogroups (*1*,*2*). Subgenogroups B4, B5, and C4 are mainly restricted to countries in Asia, whereas C1 and C2 circulate primarily in Europe and the Asia-Pacific region (*1*). Genogroup D and the newly proposed genogroup G appear to be indigenous to India, whereas genogroups E and F were recently discovered in Africa and Madagascar, respectively (*3*).

Although EV-A71 has been reported in many parts of the world, its epidemiology remains largely unexplored in Africa. An EV-A71 outbreak was documented in 2000 in Kenya, where HIV-infected orphans were infected by EV-A71 genogroup C (*4*). Several AFP cases have been associated with EV-A71 infection during 2000–2013 throughout Africa: in Democratic Republic of the Congo (*5*) (2000, n = 1); Nigeria (*6*) (2004, n = 1, genogroup E); Central African Republic (*7*) (2003, n = 1, genogroup E); Cameroon (*8*) (2008, n = 2, genogroup E); Niger (*9*) (2013, n = 1, genogroup E); and Senegal, Mauritania, and Guinea (*9*) (2013–2014, n = 3, subgenogroup C2). Four additional EV-A71 strains were obtained from captive gorillas in Cameroon during 2006–2008 (n = 2, genogroup E) (*10*) and from healthy children in Nigeria in 2014 (n = 2, genogroup E) (*11*). Molecular identification of all these isolates was based only on the analysis of sequences of the viral protein (VP) 1 capsid protein region.

Recombination events may be associated with the emergence and global expansion of new groups of EV-A71 that have induced large outbreaks of hand, foot and mouth disease with high rates of illness and death (*12*). For EV-A71, genetic exchanges have been described both within a given genogroup and with other types of enterovirus A (EV-A), usually in nonstructural genome regions P2 and P3 (*1*,*12*,*13*). However, before 2017, no complete genome sequence of EV-A71 detected in Africa has been reported, diminishing the power of such analysis. We examined the complete genome of most EV-A71 isolates reported to date in Africa to characterize the evolutionary mechanisms of genetic variability.

## The Study

We sequenced the full genome of 8 EV-A71 isolates obtained from patients with AFP (Table): isolates 14-157, 14-250, 13-365, 13-194, and 15-355 from West Africa and isolates 08-041, 08-146, and 03-008 from Central Africa. We isolated and typed these isolates as previously described (*7*–*10*) and obtained nearly complete genomic sequences using degenerated primers (*13*) and additional primers designed for gene-walking (available on request) or unbiased sequencing methods (*14*). We determined the 5′-terminal sequences by means of a RACE kit (Roche, Munich, Germany). We deposited viral genomes in GenBank (accession numbers in Table) and submitted sequence alignments under BioProject PRJNA422891. We aligned sequences using ClustalW software (http://www.clustal.org).

**Table:** Description of enterovirus isolates from patients with acute flaccid paralysis in Africa that were sequenced for characterization of enterovirus A71

Strain (reference)	Country of isolation	Patient age at diagnosis, y	Year	Virus	Genogroup or subgenogroup	Genbank accession no.
14-157 (*9*)	Senegal	3	2014	EV-A71	C2	MG672480
14-250 (*9*)	Mauritania	1.6	2014	EV-A71	C2	MG672481
13-365 (*9*)	Guinea	1.7	2013	EV-A71	C2	MG672479
15-355 (this study)	Senegal	2.4	2015	EV-A71	C2	MG013988
13-194 (*9*)	Niger	1.3	2013	EV-A71	E	MG672478
03-008 (*7*)	Central African Republic	1.9	2003	EV-A71	E	LT719068
08-146 (*8*)	Cameroon	2.6	2008	EV-A71	E	LT719066
08-041(*8*)	Cameroon	1.7	2008	EV-A71	C1	LT719067
14-254 (*15*)	Senegal	3	2014	CV-A14	NA	MG672482
*NA, not available.						

**Figure 1:**
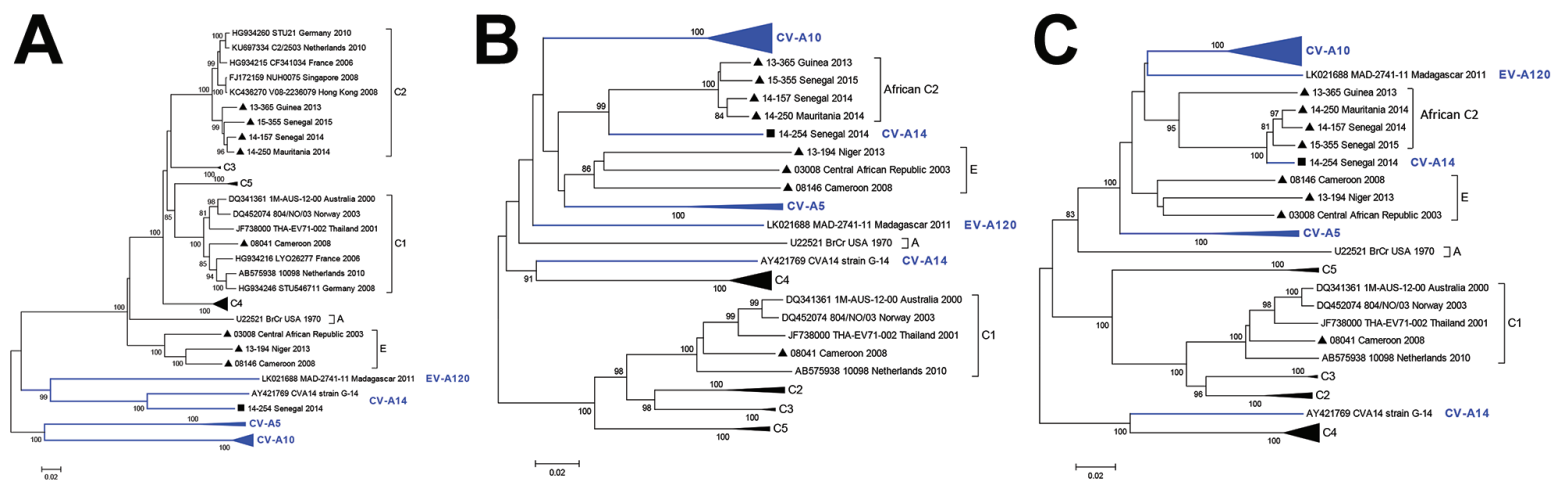
Phylogenetic relationships of the Africa enterovirus EV-A71 study strains based on A) P1 and B) P3 coding regions. An expanded version based on P1, P2, and P3 coding regions is online (LINK). Apart from the studied sequences, subgenomic datasets included their best nucleotide sequence matches identified by NCBI BLAST search (http://www.ncbi.nlm.nih.gov/BLAST) as well as representative sequences of different EV-A71 genogroups and subgenogroups originating worldwide. Trees were constructed from the nucleotide sequence alignment using MEGA 5.0. software (http://megasoftware.net/) with the neighbor-joining method. Distances were computed using the Kimura 2-parameter model. The robustness of the nodes was tested by 1,000 bootstrap replications. Bootstrap support values >75 are shown in nodes and indicate a strong support for the tree topology. For clarity, CV-A10, CV-A5, and EV-A71 subgenogroups C3, C4, and C5 have been collapsed. Study strains are indicated by laboratory code, country of origin, and year of isolation; previously published strains are indicated by GenBank accession number, isolate code, country of origin, and year of isolation. Black triangles indicate EV-A71 strains from this study; black square indicates the CV-A14 strain from this study. Strains gathered in brackets belong to EV-A71 genogroups or subgenogroups; strains marked in blue color belong to other species of EV-A. Scale bars indicate nucleotide substitutions per site. CV, coxsackievirus; EV, enterovirus.

To investigate the genetic relationship between Africa and global EV-A71 isolates, we constructed subgenomic phylogenetic trees based on the P1, P2, and P3 regions of the genome (Figure 1). We identified viral isolates showing related sequences in 1 of these 3 regions by BLAST search (http://www.ncbi.nlm.nih.gov/BLAST) and included them in the corresponding datasets used for analyses. We completed these datasets with a representative global set of EV-A71 sequences available in GenBank and belonging to the different EV-A71 genogroups (Technical Appendix). As expected, in the structural P1 region, the 8 isolates we studied clustered within their respective genogroups (C1, C2, and E), previously established by VP1-based typing (Figure 1, panel A). In particular, the isolates of genogroup E consistently clustered together (bootstrap value 100%), confirming their belonging to the EV-A71 type and their divergence from the other isolates belonging to the common genogroups A, B, and C. Analysis of the nonstructural P2 and P3 genome regions were in agreement with these data. However, the genetic heterogeneity, <12%, observed among the complete genome of genogroup E sequences highly suggested that they have circulated and diverged for years in a large geographic area in Africa. The unique Africa EV-A71-C1 strain clustered with other C1 strains originating worldwide, regardless of which genome region we analyzed. In contrast, the nonstructural sequences of Africa EV-A71 isolates of subgenogroup C2 did not cluster with their non-Africa C2 counterparts or with any of the existing EV-A71 genogroups. The incongruent phylogenetic relationships of Africa C2 strains in the different regions of the genome suggested that recombination events have occurred during evolution.

**Figure 2:**
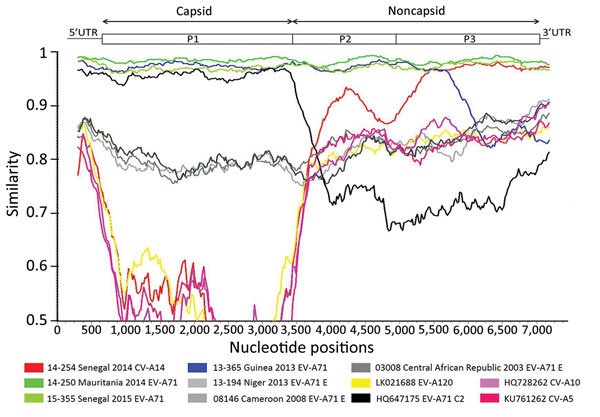
Identification of recombinant sequences in the genome of EV-A71 C2 isolates from patients with acute flaccid paralysis in Africa (14-157, 14-250, 13-365, 15-355) by similarity plot against potential parent genomes (CV-A14 strain 14-254; EV-A71 genogroup E strains 13-194, 08-146, and 03-008) and from GenBank (CV-A10, CV-A5, EV-A120). Similarity plot analysis was performed using SimPlot version 3.5.1 (http://sray.med.som.jhmi.edu/SCRoftware/simplot) on the basis of full-length genomes. For the analysis, we used a window of 600 nt moving in 20-nt steps. Approximate nt positions in the enterovirus genome are indicated. The enterovirus genetic map is shown in the top panel. We used the genome of EV-A71 study strain 14-157 as a query sequence. UTR, untranslated region.

To examine further recombination events, we analyzed EV-A71-C2 study strains by similarity plot against potential parental genomes (Figure 2). This analysis showed that sequences 14-157, 14-250, and 15-355 had high similarity (>95%). By contrast, 13-365 diverged from the other C2 isolates around nt 5600 in the P3 region, suggesting a recombination breakpoint. The analysis showed high sequence similarity (>97%) between the studied EV-A71-C2 isolates and other subgenogroup C2 strains over the P1 capsid region. Conversely, in the noncapsid region, sequence similarity between Africa EV-A71-C2 isolates and classical subgenogroup C2 isolates (e.g., GenBank accession no. HQ647175) was much lower (66%–77%). This finding confirmed a recombination event of the Africa EV-A71 C2 lineage with an unknown enterovirus, the most likely breakpoint being located between nt 3596 and 3740, within the 2A gene. Sequence identity of EV-A71-C2 study strains with their closest related viruses (coxsackievirus A10 [CV-A10], CV-A5, EV-A120, and EV-A71 genogroup E strains) in the 3′ half of the genome was <87.7%. Of note, we found much higher sequence identity with the full-genome sequence of CV-A14 isolate in our database, obtained in 2014 from a patient with AFP in Senegal (*15*). This strain features a high similarity value (>97%) with the 3′ half of the genomes of EV-A71-C2 West Africa strains (Figure 2), indicating that their P3 regions share a recent common ancestor. Because these strains belong to 2 different types, this finding strongly suggests that genetic exchanges occurred through intertypic recombination. This result cannot be a result of cross-contamination during the sequencing process because the CV-A14 and EV-A71 isolates were sequenced on 2 different platforms.

## Conclusions

Genogroup E was previously identified and characterized only on the basis of VP1 analysis (*3*). This study confirms the circulation in West and Central Africa of EV-A71 isolates belonging to the new genogroup E on the basis of the characterization of whole genomes. The divergence among isolates indicates that this genogroup has been extensively circulating in Africa. We also suggest that the common ancestor of EV-A71-C2 strains in West Africa has undergone recombination with >1 EV-A circulating in Africa. Genogroup E and recombinant C2 appear to be indigenous to Africa; they have not yet been detected elsewhere. Further exploration of environmental or clinical samples using deep sequencing technology would be of interest to determine the extent of EV-A71 circulation in Africa in the absence of AFP cases. Systematic surveillance based on full-genome sequencing could also serve to monitor these viruses for potential recombinations and to study their role in the emergence of new EV-A71 variants in Africa.

Technical Appendix More information about the enterovirus isolates used for phylogenetic analysis of enterovirus A71 in Africa.
