# Supplementary material for: Genetic Characterization of Enterovirus A71 Circulating in Africa
Source: Emerg Infect Dis. 2018 Apr;24(4):754–7. doi: 10.3201/eid2404.171783 (PMC5875259; doi:10.3201/eid2404.171783)
Supplement: Technical Appendix — More information about the enterovirus isolates used for phylogenetic analysis of enterovirus A71 in Africa. [file 17-1783-Techapp-s1.pdf]

Article DOI: https://doi.org/10.3201/eid2404.171783

Genetic Characterization of Enterovirus A71 Circulating in Africa

Technical Appendix

Technical Appendix Table. Enterovirus isolates in collapsed groups used for phylogenetic analysis of enterovirus A71

Virus	GenBank accession no.
CV-A10	KU578127
CV-A10	KY272009
CV-A10	KX595288
CV-A10	KU578135
CV-A10	KY272008
CV-A10	KP289394
CV-A10	KY272007
CV-A10	KX768156
CV-A10	KU578128
CV-A10	KU578133
CV-A10	KY272010
CV-A10	KJ641623
CV-A10	KP289395
CV-A10	KP289397
CV-A10	KP009574
CV-A10	KT588920
CV-A10	KU578130
CV-A10	KY012321
CV-A10	KX430805
CV-A10	KX430808
CV-A5	KU761262
CV-A5	KP289364
CV-A5	KP289362
CV-A5	KP289363
EV-A71 C3	DQ341355
EV-A71 C3	DQ341356
EV-A71 C4	KF543271
EV-A71 C4	KJ686306
EV-A71 C4	KU159435
EV-A71 C4	KU159434
EV-A71 C4	KT345959
EV-A71 C4	HM002485
EV-A71 C4	FJ360544
EV-A71 C4	GQ994991
EV-A71 C4	KC954663
EV-A71 C4	KP861243
EV-A71 C4	JN001860
EV-A71 C4	EU812515
EV-A71 C4	KC436272
EV-A71 C5	EU527983
EV-A71 C5	KJ686308
EV-A71 C5	KC296444

*CV, coxsackievirus; EV, enterovirus.
